# *Scedosporium* and *Lomentospora* Infections: Contemporary Microbiological Tools for the Diagnosis of Invasive Disease

**DOI:** 10.3390/jof7010023

**Published:** 2021-01-04

**Authors:** Sharon C.-A. Chen, Catriona L. Halliday, Martin Hoenigl, Oliver A. Cornely, Wieland Meyer

**Affiliations:** 1 Centre for Infectious Diseases and Microbiology Laboratory Services, Institute of Clinical Pathology and Medical Research, New South Wales Health Pathology, Westmead Hospital, Westmead, Sydney, NSW 2145, Australia; Catriona.Halliday@health.nsw.gov.au; 2 Marie Bashir Institute for Infectious Diseases & Biosecurity, The University of Sydney, Sydney, NSW 2006, Australia; wieland.meyer@sydney.edu.au; 3 Division of Infectious Diseases and Global Health, University of California San Diego, San Diego, CA 92103, USA; hoeniglmartin@gmail.com; 4 Clinical and Translational Fungal-Working Group, University of California San Diego, San Diego, CA 92103, USA; 5 Section of Infectious Diseases and Tropical Medicine, Medical University of Graz, 8036 Graz, Austria; 6 Department of Internal Medicine, Excellence Centre for Medical Mycology (ECMM), Faculty of Medicine and University Hospital Cologne, University of Cologne, 50923 Cologne, Germany; Oliver.cornely@uk-koeln.de; 7 Translational Research Cologne Excellence Cluster on Cellular Responses in Aging-associated Diseases (CECAD), 50923 Cologne, Germany; 8 Clinical Trials Centre Cologne (ZKS Koln), 50923 Cologne, Germany; 9 Molecular Mycology Research Laboratory, Centre for Infectious Diseases and Microbiology, Clinical School, Sydney Medical School, Faculty of Medicine and Health, The University of Sydney, Westmead, Sydney, NSW 2006, Australia; 10 Westmead Hospital (Research and Education Network), Westmead, NSW 2145, Australia; 11 Westmead Institute for Medical Research, Westmead, NSW 2145, Australia

**Keywords:** *Scedosporium*, *Lomentospora prolificans*, culture, histopathology, PCR-based diagnosis, MALDI-TOF MS, antifungal susceptibility, whole genome sequencing

## Abstract

*Scedosporium*/*Lomentospora* fungi are increasingly recognized pathogens. As these fungi are resistant to many antifungal agents, early diagnosis is essential for initiating targeted drug therapy. Here, we review the microbiological tools for the detection and diagnosis of invasive scedosporiosis and lomentosporiosis. Of over 10 species, *Lomentospora prolificans*, *Scedosporium apiospermum*, *S. boydii* and *S. aurantiacum* cause the majority of infections. Definitive diagnosis relies on one or more of visualization, isolation or detection of the fungus from clinical specimens by microscopy techniques, culture and molecular methods such as panfungal PCR or genus-/species-specific multiplex PCR. For isolation from respiratory tract specimens, selective media have shown improved isolation rates. Species identification is achieved by macroscopic and microscopic examination of colonies, but species should be confirmed by ITS with or without β-tubulin gene sequencing or other molecular methods. Matrix-assisted laser desorption ionization-time of flight mass spectrometry databases are improving but may need supplementation by in-house spectra for species identification. Reference broth microdilution methods is preferred for antifungal susceptibility testing. Next-generation sequencing technologies have good potential for characterization of these pathogens. Diagnosis of *Scedosporium*/*Lomentospora* infections relies on multiple approaches encompassing both phenotypic- and molecular-based methods.

## 1. Introduction

*Scedosporium* species and *Lomentospora prolificans* (formerly *Scedosporium prolificans*) [1] are increasingly appreciated causes of opportunistic fungal infections but can also act as primary pathogens [2,3]. Severely immunosuppressed individuals with hematological malignancies and/or stem cell or organ transplant recipients are at high risk for infection; however, immunocompetent individuals sustaining major trauma or near-drowning events are also at risk [2,4]. In addition, patients with chronic lung disease including cystic fibrosis (CF) are not uncommonly colonized with these fungi which may subsequently lead to invasive disease [2,3,4,5]. Clinical manifestations are protean. Disseminated disease is more common in immunocompromised patients with involvement of the lungs, brain, skin and heart whilst infections related to trauma or near-drowning events typically result in focal bone/joint and soft tissue, and lung and/or central nervous system (CNS) disease, respectively [2,3,4,6].

Many *Scedosporium*/*Lomentopsora* species are intrinsically resistant to current antifungal agents [7,8]. This, and their high mortality (>50%) [2,3,9], emphasize the importance of timely recognition of infection. The microbiology laboratory is central to assisting clinicians in diagnosis. It is thus apt to give pause to consider the armamentarium of diagnostic tools and how best to harness them for early diagnosis. As the clinical presentation and antifungal drug susceptibilities may be species dependent, we first briefly summarize the taxonomic classification of this group of pathogens to place diagnosis in context. We then review the microbiological diagnosis of infection with attention to traditional culture-based, molecular- and proteomic-based diagnostic approaches that can be utilized by clinical laboratories, and include drug susceptibility to current and newer antifungal agents. We conclude with a brief discussion of next generation sequencing (NGS) techniques in the diagnosis of infection.

## 2. Taxonomy and Species

The nomenclature of the genera *Scedosporium*/*Pseudallescheria* has undergone a number of revisions. To begin with, two species within the genus *Scedosporium*, *Scedosporium apiospermun* (with its teleomorph *Pseudoallescheria boydii*) and *Scedosporium prolificans* were recognized till the detailed application of molecular phylogenetic studies in 2005 where Gilgado at al. [10] undertook the first comprehensive review of the genus, employing four loci: β-tubulin (*BT2*,) *TUB*, calmodulin and the internal transcribed spacer regions (ITS1/2) of the rDNA gene cluster. They maintained two main “species” *Scedosporium apiospermun* (teleomorph *P. boydii*) as a species complex and *Scedosporium prolificans.* In addition, two new species *S. aurantiacum* and *S. minutisporum* were described. In a follow up study [11], the distinction between *S. apiospermum* and *P. boydii* was crystallized, and an additional new species, *S. dehoogii*, described. Further new species have since been assigned, e.g., *S. deficiens* (closely related to *S. dehoogi)*, *S. cereisporum* (closely related to *S. aurantiacum*) [12,13].

**Figure 1:**
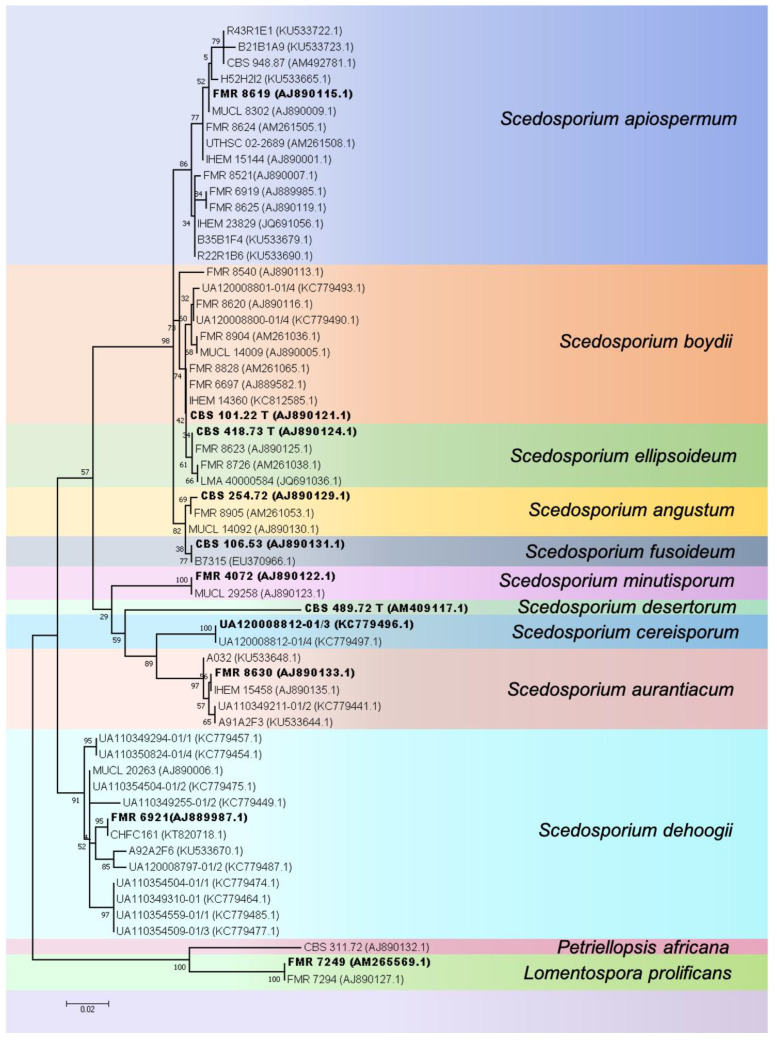
Phylogenetic tree of *Scedosporium* species based on 60 tubulin sequences (*TUB*, exon 5 and 6), representing the known genetic variation within the genus, using Maximum Likelihood with the K2+G model and 500 bootstrap replications. Bootstrap values >50 are given. The tree is rooted with the outgroup species *Petriellopsis africana* and *Lomentospora prolificans*. GenBank accession numbers are given in brackets behind the strain numbers. Bold font indicates sequences of type strains.

With the establishment of the “One Fungus = One Name” rule in fungal taxonomy which allows only a single name per fungus [14] the genus name “*Scedosporium*” was retained at the expense of “*Pseudallescheria*. [1]. Currently, the genus *Scedosporium* contains at least 10 species (*S. angustum, S. aurantiacum, S. apiospermum, S. boydii, S. cereisporum, S. dehoogii, S. desertorum, S. ellipsoideum, S. fusoideum*, and *S. minutisporium*)*,* with *S. angustum, S. apiospermum, S. boydii, S. ellipsoideum* and *S. fusoideum* considered a species complex [2]. *S. apiospermum, S. boydii* and *S. dehoogii* show extensive genetic diversity (see Figure 1 for an explanatory phylogenetic tree), suggesting the presence of cryptic species. Notably, *Scedosporium prolificans* was considered only distantly related to *Scedosporium* and was re-classified as “*Lomentospora prolificans”,* and the genus *Lomentospora* re-instated for this species [1].

Of the species that cause disease in humans, four—*Scedosporium boydii*, *S. apiospermum*, *S. aurantiacum* and *L. prolificans*—account for the large majority of infections. Species-specific virulence, ecological, and clinical differences are described elsewhere [2,3,15,16,17] and are beyond the scope of the present review. *L. prolificans* is the only *Lomentospora* species of note that causes human disease.

## 3. Microbiological Diagnosis

Despite advances in non-culture-based diagnostic methods for scedosporiosis, the detection of the causative fungal agent from clinical samples by direct microscopic examination of fresh specimens, or by histopathological techniques, together with culture on mycological media remains paramount. Indeed, the 2019 updated European Organization for Research and Treatment of Cancer/Mycoses Study Group Education and Research Consortium (EORTC/MSGERC) definitions for invasive fungal disease (IFD) emphasize the visualization of fungal hyphae and/or culture of the fungus from an affected site as a criterion for proven fungal infection [18].

Molecular techniques are increasingly used to directly detect *Sceodosporium/Lomentospora* DNA in clinical specimens followed, typically by DNA sequencing or barcoding to identify the causative species. The use of proteomic approaches, e.g., matrix-assisted laser desorption ionization time-of-flight mass spectrometry (MALDI-TOF MS) have replaced phenotypic methods as a first line for species identification in many laboratories, backed up by molecular approaches. At present, serological methods have little role in routine diagnosis.

Finally, albeit not within scope of the present review, microbiological diagnosis should always be accompanied by imaging as appropriate to confirm diagnosis and determine the extent of infection. Recently, guidance on how to diagnose rare mold infections, including those by *Sceodosporium/Lomentospora* has been issued by the European Confederation of Medical Mycology in cooperation with the International Society of Human and Animal Mycoses and the American Society of Microbiology [19]. This document includes detailed recommendations on imaging.

### 3.1. Histopathology

Whilst the inability of histopathological methods to recognize species is acknowledged, examination by traditional stains such as the Gomori methenamie silver (GMS), hematoxylin and eosin (H & E) and periodic acid-Schiff (PAS) stains for the presence of fungal elements has good utility to provide a presumptive diagnosis of any IFD in a cost-effective manner [20]. Many experts strongly recommend such histopathological examination of affected tissue and other clinical specimens where there is a high index of suspicion for *Scedosporium*/*Lomentospora* infection. Expertise is required to distinguish the features described below.

Hyphae of *Scedosporium* spp. are hyaline in appearance and when visualized, confirm the presence of fungal infection, but are non-specific, and it is not possible to distinguish these from the hyphae of other hyaline molds such as *Aspergillus*, *Fusarium* and *Paecilomyces* spp. Nonetheless, the hyphae, typically located within areas of necrosis, are septate, non-pigmented and may show acute-angle branching. More often than not, they appear irregular, sometimes with branches bridging two parallel hyphae to form an H-shaped pattern; the last is considered by some experts to be highly suggestive of *Scedosporium* and *L. prolificans* [21]. Of note, the hyphae of *L. prolificans* are often, though not always, melanized [20,21]. In GMS-stained sections, lemon-shaped conidia measuring 5 × 7 µm in size may be seen, located terminally or laterally on the hyphae. Intravascular conidiation or conidiation, with purple-colored conidia, within tissue may also be evident [2,21]. Thrombosis of blood vessels is usual. In brain tissue, granulomatous inflammation with multinucleate giant cells and hyphae within the cerebral vasculature are described [22]. Whilst in situ hybridization with a *Scedosporium*-specific DNA probe has been reported [23], its utility in routine diagnostics is uncertain.

### 3.2. Microscopy and Culture

Despite the fact that microscopy and culture are insensitive and slow (2–10 days), their application to clinical specimens remains a cornerstone of diagnosis. Further, culture yields an isolate for drug susceptibility testing.

These fungi can be cultured from any clinical specimen including tissue, respiratory tract specimens, eye, skin and blood. Both *Scedosporium* spp. and *L. prolificans* will grow in standard blood culture systems although most publications describe the recovery of *L. prolificans* from blood cultures (summarized in 2, 3, 6). More often, however, these fungi are recovered from sputum and respiratory tract samples including bronchoalveolar lavage (BAL) fluid. Isolates from sterile sites should always be considered clinically significant. Direct microscopy of clinical specimens can rapidly suggest a diagnosis in appropriate clinical context. Yeast-to-hyphal structures may be seen on Gram stain of blood cultures. There are few data on use of fluorescent brighteners or potassium hydroxide (KOH) wet mounts, the latter of which is limited to skin and corneal scrapes.

#### 3.2.1. Culture

*Scedosporium*/*Lomentospora* fungi will grow without difficulty on standard mycological media such as Sabouraud’s dextrose agar (SDA) or potato dextrose agar (PDA). However, their recovery especially from non-sterile respiratory tract specimens may be hampered by competing growth from other fast-growing molds, most often *Aspergillus* spp., leading to “missed” detection. It is also important to note that the growth of *L. prolificans*, but not *Scedosporium* spp., is inhibited by the presence of cycloheximide, if added to mycological media [3].

Hence, many laboratory mycologists choose to employ the use of semi-selective or selective media to “select” for growth of *Scedosporium*/*Lomentospora* spp. This involves the incorporation of an inhibitor, such as benomyl, cycloheximide (*Scedosporium* spp.) or dichloran (2,6-dichlro-4-nitroaniline) to inhibit non-*Scedosporium*/*Lomentospora* fungi into a base medium to reduce growth of other molds. Such media include *Scedosporium* Selective agar (SceSel+) and its newer variation Scedo-Select III which contains both benomyl and dichloran [24,25] which is preferred but these must be freshly prepared and are not available commercially. Alternatives are Dicloran Rose Bengal Chloramphenicol medium (DRBC, ThermoScientific, Oxoid, UK), which also contains benomyl [26], and Mycosel^TM^ agar (BBL, BD, USA), both of which may be purchased although the latter was found in one study to be inferior to SceSel+ agar for recovery of *Scedosporium* from sputum [27]. Most experts recommend the combination of non-selective media, e.g., SDA with selective media, preferably SceSel+ or DRBC. Fungal colonies grow within 48–72 h and then are subcultured onto PDA at 25 °C for 10–14 days, to enable slide cultures for delineating microscopic characteristics of colonies.

Although many laboratories now rely on molecular-based methods to confirm species identification, there are phenotypic features from cultured colonies that can assist identification, briefly summarized in Section 3.2.1.1 and Section 3.2.1.2 below. Key macroscopic and microscopic features of the major medically important *Scedosporium*/*Lomentopsora* species are given in the tables below.

##### 3.2.1.1. Macroscopic Features

**Figure 2:**
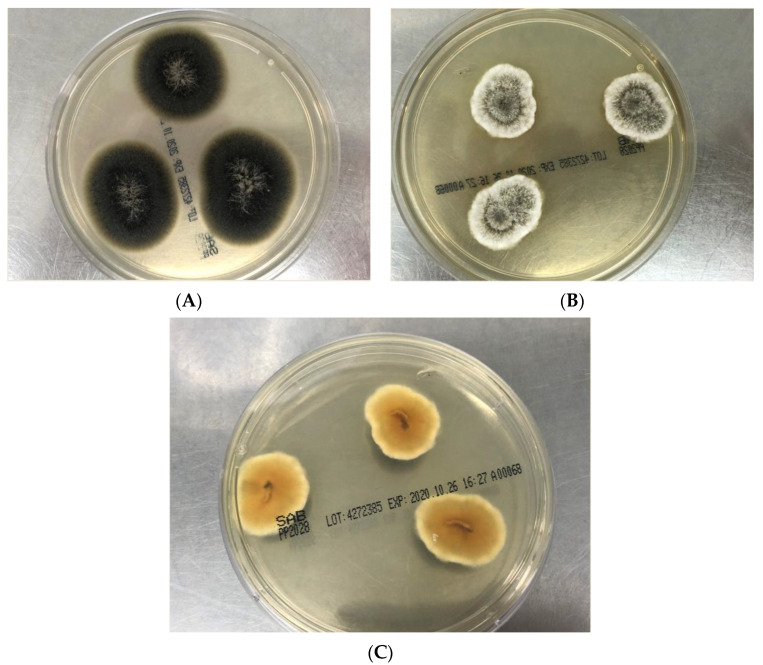
(**A**): Flat, spreading olive-grey to black colored and suede-like surface (obverse view) of *Lomentospora prolificans* on Sabouraud dextrose agar (SDA) after four days growth at 28 °C. (**B**): Greyish-white cottony to woolly surface with concentric growth pattern and white margins (obverse view) of *Scedosporium aurantiacum* on SDA after 3 days growth at 28 °C. (**C**): Brown orange center with pale yellow pigment (reverse view) of *Scedosporium aurantiacum* on potato dextrose agar after 3 days incubation at 28 °C.

Most *Scedosporium* spp. and *L. prolificans* will be sufficiently mature in growth on PDA to allow elucation of macroscopic and microscopic characteristics. One distinguishing feature of *L. prolificans* from *Scedosporium* species is its grey-back color (Figure 2A) and its susceptibility to cycloheximide [3]. The variation of colony color of *S. apiospermum*, *S. boydii* and *S. aurantiacum* are given in Table 1. Colonies of *S. aurantiacum* are often seen in a pattern of concentric growth (representing aerial mycelium, Figure 2B), which varies from yellow to gray/brown in color; further, many isolates produce a distinctive yellow diffusible pigment on PDA after few days’ incubation (Figure 2C). *S. aurantiacum* is also able to grow at 45 °C. Table 1 shows that the colony characteristics of *S. apiospermum* and *S. boydii* are very similar if not identical and these species are unable to be distinguished from each other [11].

##### 3.2.1.2. Microscopic Features

**Figure 3:**
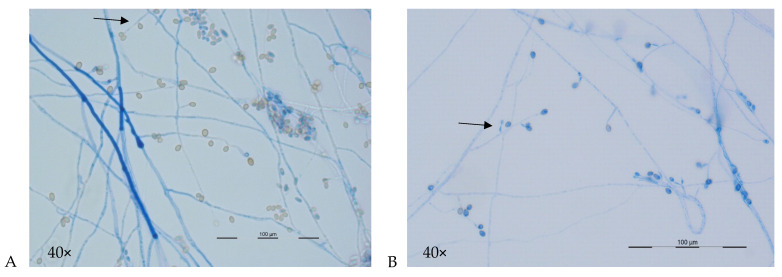
A and B: Microscopic morphology of Scedosporium aurantiacum (**A**) and Lomentospora prolificans (**B**) 40x magnification. (**A**) Hyphae are septate with simple long conidiophores (arrow) bearing conidia singly or in small groups. Conidia (4–9 × 6–10 μm) are single-celled, pale brown ovoid in shape and are rounded above with truncated bases. (**B**) Hyphae are hyaline and septate with conidia born in small groups on basally swollen, flask-shaped conidiophores (arrow). Conidia (2–5 × 3–7 μm) are hyaline to pale brown, one-celled and oval-shaped with smooth walls.

Microscopic features may suggest the presence of *L. prolificans* and disitinguish it from *Scedosporium* spp., notably the visualization of flask-shaped conidiophores which are inflated or swollen at the base, from which single, or clusters of conidia emerge (Figure 3A,B). Species distinction within the *S. apiospermum* species complex is not possible (Table 2). The teleomorph state (where known) and characteristics of cleistothecia (or closed spore-bearing structures), ascopores and conidia (shape and wall thickness) for the major species are shown in Table 2 with detailed descriptions elsewhere [2,11,28]. For *S. aurantiacum*, coniodiophores are solitary and borne on aerial mycelium. Conidia are typically smooth, and thick walled, and often obovoid in shape [2,28].

### 3.3. Species Identification

#### 3.3.1. Phenotypic Methods

Phenotypic identification methods include growth at 40 °C and 45 °C (Table 1), pattern of utilization of carbohydrates and enzymatic activities. Although collectively these can aid in species differentiation, they are not able to separate species within the *S. apiospermum* complex. In some laboratories, time-consuming “in-house” systems are used. Commercial kits for species identification include the Taxa Profile Micronaut^TM^ (Marlin Diagnostika GmbH, Berlin, Germany), a system examining various physiological reactions of these fungi [29], and the GEN III MicroPlate^TM^ (Biolog Inc., Hayward, CA, USA) [30]. However, the latter was set up to distinguish between high- and low-virulent strains of *S. aurantiacum* rather than for species identification. In practice, phenotypic methods are largely superseded by MALDI-TOF MS and molecular approaches.

#### 3.3.2. MALDI-TOF MS

Although multi-locus DNA sequencing is considered the reference method (see later), MALDI-TOF MS offers good potential for fast, and relatively economical identification of *Scedosporium* species and *L. prolificans* with comparable accuracy to many molecular methods with the *proviso* that MS databases contain reference spectra [31]. The standardisation of growth conditions for fungal protein extraction and of methods for extended sample preparation to achieve good quality mass spectra is also essential [32]. The Vitek^®^ MS v3.0 (bioMerieux, Marcy L’Etoile, France) database was approved by the US Food and Drug Administration (FDA) for identification of molds in July 2017, but does not contain spectra of *S. aurantiacum*. Consequently, within the *S. apiospermum* complex, only *S. apiospermum* and *S. boydii* can be identified by the Vitek^®^ MS v 3.0 system [33]. The other commercial MALDI-TOF MS systems (MALDI Biotyper (Bruker Daltonics, Bremen, Germany), Andromas (Andromas SAS, Paris, France) and Axima@SARAMIS (Shimadzu/AnagnosTec, Duisburg, Germany)) are also inadequate for the identification of *Scedosporium/Lomentospora* when relying on their database alone [34,35,36]. As such, the success of MALDI-TOF MS is enabled by complementation of commercial databases by in-house libraries together with consistency of sample preparation methods used to generate these mass spectra [32,37].

Sleiman et al. [34] reported a 5-fold improvement (18 to 94%, *n* = 17) in the identification of *Scedosporium/Lomentospora* isolates (score values ≥ 2.00) after the Bruker Filamentous Fungi Library v1.0 (Bruker Daltoniks) was supplemented with an in-house database. Although *L. prolificans* was represented in the Bruker database, only one of four strains was identified, yet all four were identified using the enhanced database, suggesting test performance also relies on the inclusion of spectra from local strains in the database. In another study, only one of 21 *Scedosporium* spp. isolates were correctly identified to genus level using the same Bruker library. The inclusion of 24 reference spectra from *S. boydii*, *S. apiopsermum* and *L. prolificans* to an in-house database allowed 76% (16/21) of isolates to be identified to species level but with scores of 1.65–1.9 [38].

Of note, Sitterle et al. [36] built a reference database comprising *L. prolificans* and five morphologically indistinguishable species—*S. boydii, S. apiosperum, S. aurantiacum, P. minutispora* and *S. dehoogii* for use on the Andromas platform. The database was evaluated against 64 isolates previously identified by multi-locus sequence type (MLST) analysis, and all isolates were unequivocally identified in minutes (vs. several days for MLST), demonstrating the power of MALDI-TOF MS with a comprehensive database. However, a similar study [35] using reference spectra from an online database was unable to distinguish within the *S. apiospermum* complex.

#### 3.3.3. Identification by Molecular Methods (Isolates)

DNA sequence-based analysis is the current “gold standard” for species identification of all fungi. Sequencing of the primary fungal DNA barcode, the ITS region is able to identify the main pathogenic species of *Scedosporium*/*Lomentospora* [39]; however, for very closely related species, e.g., *S. boydii* and *S. ellipsoidea* (an environmental species) [1], amplification of an additional locus, the partial *β-tubulin* gene (*βT2*) may be required to resolve species. Of central importance is a reliable reference sequence database such as those of the Westerdijk Institute (http://www.westerdijkinstitute.nl/Collections/BioloMICSSequences.aspx) or the International Society for Human and Animal Mycology (http://its.mycologylab.org) for sequence pairwise alignment.

Other than DNA sequencing, the analysis of repetitive DNA sequences of isolates (rep-PCR) using the Diversilab^TM^ System (bioMerieux) enabled not only the identification of a number of *Scedosporium* species but also identified genotypes within species [40]; of a total of 63 isolates, rep-PCR identified eight genotypes each of *S. apiospermum* and *S. boydii* whilst all *S. aurantiacum* isolates shared the same profile. Rep-PCR profiles were concordant with their MLST (see below).

To this end, species identification may also be undertaken by MLST as inferred in Section 3.3.2 with at least three web-based MLST schemes available for comparison of MLST genotypes—one each for *S. apiospermum*, *S. boydii* and *S. aurantiacum* (https://mlst.mycologylab.org) [41]. However, MLST does not readily translate to routine use in diagnostic laboratories and requires expertise for set up and interpretation.

Finally, rolling circle amplification (RCA) which uses a simple isothermal amplification format can differentiate between sequences to a single nucleotide level [42]. Using an ITS-directed RCA to identify *Scedosporium* species, Zhou et al. distinguished between *L. prolificans* and *S. apiospermum*/*S. boydii* [43]. The advantages of RCA are that procedures can be performed rapidly (within 2 h) and with a simple heating block or water bath, with simplicity and cost effectiveness.

### 3.4. Direct Detection in Clinical Specimens by Molecular Methods

The high resolving power together with increasing access to molecular biology technologies in clinical laboratories have enhanced the laboratory’s capacity to rapidly detect and identify *Scedosporium*/*Lomentospora* fungi directly from clinical specimens. Such approaches include broad range PCR followed up by DNA sequencing and species-specific multiplex PCR in various formats including real time PCR and oligonucleotide arrays. Each has its relative strengths and uptake of technology is dependent on laboratory resources. There are no standadized commercial assays.

#### 3.4.1. Broad Range PCR

Broad range or panfungal PCR is increasingly employed in clinical laboratories to identify fungi directly from fresh and formalin fixed paraffin-embedded (FFPE) tissue as well as from other types of clinical specimens including BAL fluid and cerebrospinal fluid (CSF). Multiple assays have been published [44,45,46]. Most often, the ITS regions (ITS1, ITS2 or both) or the 28S rDNA locus are amplified using universal fungal primers [47] followed by DNA sequencing [48], although high-resolution melt curve analysis by real-time PCR is increasing [49,50,51]. Species identification of by sequencing would follow the same principles as those of cultured isolates (Section 3.3.3). In general, accuracy and specificity are good although sensitivity varies with specimen type.

**Figure 4:**
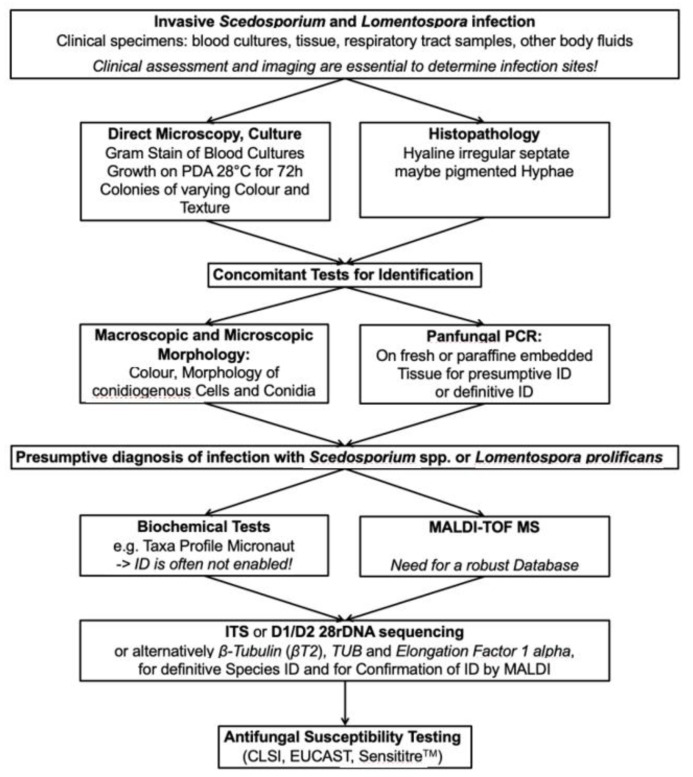
Workflow for diagnosis of invasive *Scedosporium* and *Lomentospora prolificans* infections. CLSI = Clinical and Laboratory Standards Institute; EUCAST = European Committee on Antimicrobial Susceptibility Testing; rDNA = ribosomal DNA; ID = identification; ITS = internal transcribed spacer; MALDI-TOF MS = matrix-assisted laser desorption/ionization time-of-flight mass spectrometry; PCR = polymerase chain reaction; PDA = Potato dextrose agar; *TUB* = Tubulin.

In one report, using a panfungal real-time PCR, test performance was best when used on sterile body fluids (sensitivity 100%, specificity 96%, negative predictive value (NPV) 100% and positive predictive value (PPV) 86%)) but performance was considerably lower when performed on BAL fluid (90%, 75%, 86% and 82%, respectively) [51]. Gomez et al. using a sequence-based PCR observed a sensitivity and specificity of 96.1% and 98.2%, respectively, in patients with proven IFD (*n* = 60). Assay sensitivity was similar across different specimen types (100% for fresh tissue, 100% for sterile body fluids and 90% for FFPE) [46]. These two sets of findings saw a greater sensitivity than that reported by others [48,52] where using an ITS-1 targeted panfungal PCR Lau et al. [48] made the point that sensitivity is highest when fungal elements were visible in fresh or histopathological sections. Assay sensitivity, which included the detection of *L. prolificans* in tissue, was 97% when applied to fresh tissue and 68% when performed on FFPE tissue [48]. For 20 culture-negative but histopathology-positive specimens reported by Trubiano and colleagues, diagnosis of IFD to causative species level by panfungal PCR occurred in 35% (6/20) [52]. In support of the above, the 2019 updated EORTC/MSGERC IFD definitions recommend the amplification of fungal DNA by PCR combined with DNA sequencing only when fungal elements are seen on histopathology specimens or sterile body fluids [18]. Under these circumstances, the assay has good utility and value adds to the diagnostic approach (Figure 4).

**Figure 5:**
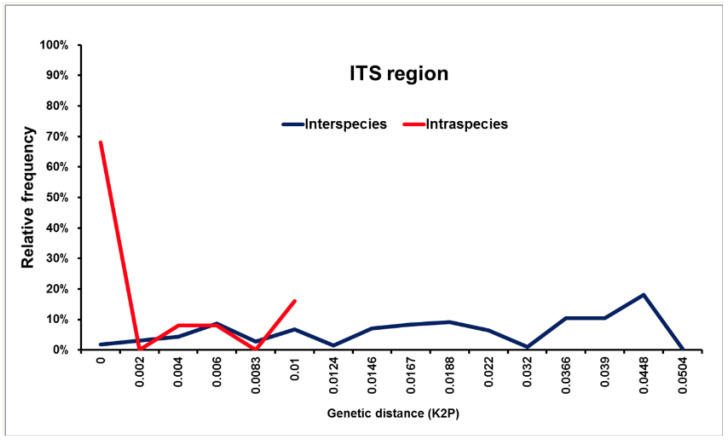
Distribution of interspecies (blue line) and intraspecies (red line) pairwise Kimura 2-parameter genetic distances in the genus *Scedosporium* based on the primary fungal DNA barcode the ITS1/2 region, based on 60 ITS1/2 sequences of *Scedosporium angustum, Scedosporium aurantiacum, Scedosporium apiospermum, Scedosporium boydii, Scedosporium cereisporum, Scedosporium dehoogii, Scedosporium desertorum, Scedosporium ellipsoideum, Scedosporium fusoideum* and *Scedosporium minutisporum.* No DNA barcoding gap was identified using the ITS1/2 region. All taxa were subjected to pairwise sequence divergence calculations using the Kimura 2-parametric distance model (K2P) in MEGA ver. 5.2.2.

Of note, ITS-directed assays may not be able to distinguish between species within the *S. apiospermum* complex, as no DNA barcoding gap is present, due to the relatively large degree of genetic variation found in this species complex. The use of alternative loci, such as the *βT2* gene, *TUB* and elongation factor 1 alpha genes may be necessary (see Figure 5).

Conversely, the interpretation of broad range PCR results when performed on non-sterile specimens especially on BAL fluid is problematic and PCR-positive results cannot discriminate between lung infection and airway colonization. Sensitivity of panfungal PCR on BAL fluid is also lower in patients receiving mold-active therapies [52].

Finally, Salehi et al. used multiple real-time quantitative PCR assay targeting the ITS2 region to identify *Scedosporium*/*L. prolificans* in a variety of FFPE specimens where fungal elements were seen. [50]. Probes used included a pan-“Scedosporium” probe, and one each for *S. apiospermum*, *L. prolificans* and *S. aurantiacum*. The study was limited in that only a single specimen was identified as containing DNA from these fungi.

#### 3.4.2. Targeted Multiplex PCR

There are relatively few reports on the use of multiplex PCR for the specific detection of *Scedosporium*/*Lomentopsora* in clinical specimens. Using species-specific ITS-directed primers as well as those targeting the *28S* rDNA gene, one study used standard multiplex PCR to detect *L. prolificans*, *S. apiospermum* complex and *S. aurantiacum* on DNA extracts from stored sputum specimens from CF patients, in comparison with culture. The PCR yielded a positive result in 18/29 samples from 7/11 (63.6%) patients; the test specificity was 97.2% with a PPV of 78.3% and NPV of 94.1% [53]. Insufficient target DNA and PCR inhibition can limit test sensitivty. Although simple, at present, direct muitpelx PCR should complement culture, rather than replace it.

Mulitplex PCRs may also be in real time format using fluorescent-labelled primers, probes or dyes. Experience with such PCR assays to detect and identify *Scedosporium* species in routine diagnostics is limited. Castelli et al. developed two separate ITS-targeted PCR assays for species identification of *L. prolificans* and *S. apiospermum* and tested their assay in infected mice. In mice infected with *L. prolificans*, the assay sensitivity was 95.5% for lung tissue, 85% for serum and 83.3% for whole blood. For *S. apiospermum* infected mice the sensitivity of the assay was 97.2%, 81.8% and 54.5% for lung, serum and blood, respectively [54].

#### 3.4.3. Oligonucleotide Array Assay

Bouchara et al. [55] were one of the first to apply this technology to direcly detect fungi including *Scedosporium* in the sputum of CF patients. For 57 sputa, results were compared with those of culture. In more than half the cases (*n* = 33 samples), the array detected a more diverse range of fungi than culture. *Scedosporium* fungi were identified but not at species level. Disadvantages include the high costs for set up and need for batch testing for efficiencies.

### 3.5. Serology

Diagnosis by serological approaches have encompassed the study of both panfungal biomarkers as well as *Scedosporium*/*Lomentospora*-specific markers. However, large scale studies on their diagnostic utility remain sparse. The best studied panfungal marker is 1,3-β-D-glucan (BDG) which is found in the cell wall of fungi and can be detcted in the blood of patients with IFDs except in Mucorales and *Cryptococcus* infections. The clinical utility of this test is well summarized by meta-analyses [56,57] and demonstrates generally good sensitivity and specificty (for any IFD) in various patient populations. This has typically included a small number of patients with *Scedosporium* and *L. prolificans* infections and is supported by the detection of BDG in serum in reports of *Scedosporium* brain abscess and *L. prolificans* fungemia [58,59,60]. The serum BDG test may thus be a useful diagnostic adjunct. Most studies have employed the Fungitell^TM^ assay (Associates of Cape Cod inc.; Falmouth, MA, USA).

There are no comemrcial *Scedosporium* or *L. prolificans*-specific serological tests. Nonetheless, Thornton and colleagues have develoepd a number of *Scedosproium-Lomentopsora* specific monoclonal antibodies (Mabs) that enable species identification. One targets an extracellular 120-kDa antigen located in the spore and hyphal walls of *S. apiospermum* and *S. boydii* whilst the other tragets the enyme tetrahydroxynaphthalene reductase which plays a role in the biosysnthesis of melanin in *L. prolificans* [61,62]. Certain *Scedosporium* proteins such as cytosolic catalase have been studied as markers of infection as have various metabolites and siderophores [63,64] of these fungi, but currently these markers are limited to research laboratories. Mina et al. reported that sera of CF patients with *S. apiospermum* complex infection demonstrated antibodies to catalase A1 of *S. boydii* by ELISA whilst those colonized or infected with *Aspergillus fumigatus* did not [63]. Study in different patient cohorts is warranted to investigate the role of novel antigen/antibody tests in serodiagnosis.

### 3.6. In Vitro Susceptibility to Antifungal Agents

*Scedosporium*/*Lomentospora* fungi are resistant to many antifungal agents currently available. As there are species-specific susceptibility patterns, it is imperative the causative species is accurately identified. Further within species, minimum inhibitory concentrations (MICs) to some antifungal drugs vary between isolates, rendering it advisable to determine drug MICs to all clinically significant isolates. The methodology chosen should be an established method such as the reference broth microdilution methods of the Clinical Laboratory Standards Institute (CLSI), USA and the European Committee on Antimicrobial Susceptibility Testing (EUCAST; www.eucast.org). Where the commercial Sensititre^®^ Y010 panels are used (Trek Diagnostics Systems, ThermoFisher, Waltham, MA, USA).), comparative evaluations are recommended but in general, similar MIC results have been obtained [65,66,67].

All *Scedosporium* species and *L. prolificans* are resistant to amphotericin B (MIC_90_ generally 8—>32 mg/L), 5-flucytosine (MIC_90_ > 16 mg/L), fluconazole (MIC_90_ > 16 mg/L) and itraconazole (16—>16 mg/L) although a number of studies have observed that *S. apiospermum* complex isolates may exhibit lower MICs to itraconazole (MIC 0.5–1 mg/L) [8,66,67]. In addition, both genera have reduced susceptibility to the echinocandins with wide ranging MICs (MICs 0.006–32 mg/L) reported, with *L. prolificans* being the most “resistant’ (MICs 4–16 mg/L) [8,67]. However, for voriconazole, geometric mean (GM) MICs are relatively low for *S. apiopermum* and *S. boydii* (GM MIC 1.64 and 0.75 mg/L respectively) with similar observations for posconazole. Notably, *L. prolificans* is pan-antifungal resistant [2,8,66,68].

*L. prolificans* is also resistant to isavuconazole (MICs typcially > 4 mg/L), [8,66,68,69], however Trovato et al. observed lower MICs for isavuconazole among 27 *S. apiospermum* complex strains using both the EUCAST E. Def. 9.3 (GM MIC 1.22 mg/L) and the Etest (Liofilchem, TE, Italy; GM MIC 1.08 mg/L) [70]. Terbinafine MICs also vary with species between 0.06–32, being lowest for *S. apiospermum* [67]. Although terbinafine is not a drug that is used alone for treatment of *L. prolificans* infections, it demonstrates in vitro synergy with voriconazole and to a lesser extent with posaconazole and is used in combination typically with voriconazole [2,71,72]. The treatment of infections caused by these fungi are beyond the scope of this review but can be found in recent management guidelines [19,72].

#### New Antifungal Drugs in the Pipeline

It is pertinent to briefly touch on a number promising novel antifungal drugs for treatment of *Scedosporium* and *L. prolificans* infections. In due course, it could be argued that clinical laboratories will consider the inclusion of these agents in their antifungal susceptibility testing menu.

Fosmanogepix (formerly APX001 and E1211) is a new first-in-class antifungal with broad spectrum activity which targets the inositol acylation step in the biosynthesis of the glycosyl phosphatidyl inositol (GPI) anchor, which in turn anchors proteins to the cell wall [73]. In vitro testing of *S. apiospermum* (*n* = 28), *S. aurantiacum* (*n* = 7) and *L. prolificans* (*n* = 28) revealed that MICs were at least 10-fold lower than found for standard antifungal drugs including voriconazole [74]. The MEC_90_ values for fosmanogepix against both *S. apiospermum* and *L. prolificans* were 0.12 mg/L [74]. Another particularly promising anti-*Scedosporium* and *L. prolificans* agent is Olorofim (previously F901318). Olorofim is a first-in-class antifungal drug that inhibits dihydroorotate dehydrogenase, an enzyme central to fungal pyrimidine biosynthesis [75]. Biswas et al. tested 50 strains encompassing the four main species with MICs ranging between 0.125–0.5 mg/L [8]; similar results were reported in a US study (MIC range <0.008–0.25 mg/L) with *L. prolificans* strains having MICs at the upper end of the range [76]. Finally, *N*-chlorotaurine (NCT) has broad spectrum antimicrobial killing activity due to its transhalogenated nature, which markedly enhances its in vivo activity. Clinical phase I and II studies have demonstrated good tolerability of topical 1% NCT in aqueous solution including during inhalation [77]. Whether these results can be translated into treatment of scedosporiosis in the lung is not yet known.

### 3.7. Diagnostic Algorithms in the Microbiology and Pathology Laboratories

Given the many options for laboratory diagnosis, a systematic guide to assist laboratories with diagnosis is helpful. Whether a diagnostic approach is performed will be partially dependent on access, resources and on the size and nature of the laboratory (e.g., a reference mycology laboratory vs. a smaller district hospital). Figure 4 summarizes a diagnostic pathway for both invasive infections due to *Scedosporium* spp. and *L. prolificans*. To re-emphasize, imaging procedures are essential parallel investigations. The Global guideline for the diagnosis and management of rare mold infections refer to the strength of recommendations for each diagnostic option and give the rationale behind these recommendations [19].

## 4. Next Generation Sequencing Approaches

Given the increasingly emergent nature of *Scedosporium*/*Lomentospora* infections, the publishing of the draft genomes, using next generation sequencing (NGS) methodology, of all four major pathogenic species is welcomed [78,79,80,81]. Both short read sequencing using Illumina technology (Illumina Inc., San Diego, CA, USA) and long read sequencing for additional coverage using Oxford Nanopore Technology with the MinION (ONT, Didcot, Oxford, UK) [78] have been used. Genome sizes are similar and typical for a filamentous fungus, very large (37.6 Mb, 43.3 Mb, 43.4 Mb and 39.9 Mb for *L. prolificans*, *S. boydii, S*. *apiospermum* and *S. aurantiacum*, respectively) [78,79,80,81]. Genomes have been annotated or genes, predicted. Chromosomal analysis has revealed that *L. prolificans* contains at least 7, and possibly 11 chromosomes [78]. Of note, analysis has been undertaken using different bioinformatic tools and more study is required to understand the comparability of these results. The clinical application of NGS of *Scedosporium*/*Lomentospora* isolates using these draft genomes as a “reference” is ongoing.

As such, there are few data on the use of NGS to study genomic relationships of clinical strains and in the tracking of infection. One small study investigated a possible link between four *L. prolificans* isolates from infections occurring over an 8-month period a single hospital unit [82]. There were >10,000 single nucleotide polymorphisms (SNPs) between isolates. However, there were sparse details given as to how analysis was undertaken to for SNP calling, and the results were inconclusive to establish a genetic relationship. Study of larger numbers of isolates of each of these species in clinical context is required to determine the genetic variation as occurs in cases of sporadic infection to inform relatedness in event of case clusters. Results analyzed by different bioinformatic pipelines will influence interpretation and it is also important the ploidy of these fungi are taken into consideration when studying genetic polymorphisms.

## 5. Conclusions

In conclusion, the microbiology-pathology laboratory has good potential to assist with diagnosis of *Scedosporium* and *L. prolificans* infections. With wider access to molecular detection and identification methods, it is tempting for laboratories to reduce focus on culture and conventional identification methods as these are time consuming and require trained personnel. However, in many resource-limited settings, culture and phenotypic approaches remain mainstay of diagnosis; in this context, access to MALDI-TOF MS and molecular-based methods are a priority. It should be noted, however, that basic biology and morphology is still required for characterization of fungi and represents a criterion for the lodging of authenticated fungal strains in a recognized and curated culture collection. Hence it is important for laboratories to maintain an understanding of all diagnostic approaches.

The role of NGS genomics is currently still largely research based. Improved and standardized approaches to bioinformatic analysis of data would help position this technology into being translated into routine use.

## Figures and Tables

**Table 1:** Colony morphology, color, and growth characteristics of *Scedosporium* and *Lomentospora prolificans* on potato dextrose agar [11,28].

Species	Colony Morphology on PDA (25 °C) ^1^			Growth Temperature	
	Color (Obverse)	Color (Reverse)	Texture	40 °C	45 °C
*Lomentospora* *prolificans*	Olive-gray to black; white colored tufts (mycelium)	Dark brown or gray, almost black	Downy to cottony	Yes	Variable
*Scedosporium* *apiospermum*	White gray, becoming darker gray or brown	Dark brown or gray, almost black	Downy to cottony	Yes	No
*Scedosporium boydii*	White gray, becoming darker gray or brown	Dark brown or gray, almost black	Downy to cottony	Yes	No
*Scedosporium* *aurantiacum*	Yellow gray to brown gray; concentric growth pattern; white margin	Brown orange or brown but may be colorless	Dense, cottony to woolly	Yes	Yes

^1^ PDA, potato dextrose agar.

**Table 2:** Microscopic features of Scedosporium species and Lomentospora prolificans.

Species	Cleistothecia	Ascospores	Conidiogenous Cells		Conidia	
			Shape	Distribution	Shape	Size
*Lomentospora prolificans*	No Teleomorph		Flask-shaped with basal swelling	Solitary or on branched conidiophores	Globose to subglobose, thick-walled	3 × 5 µm
*Scedosporium apiospermum*	*P. apiosperma* (previously *P. boydii)*		Cylindrical	Solitary or on branched conidiophores	Globose to subglobose, thick- walled	5–7 × 4–6 µm
*Scedosporium boydii*	Globose to subglobose, 100–300 µm diameter	Broadly fusiform, pale yellow or brown—copper; 6–9 × 5–6 µm	Cylindrical	On branched coniodiophores or solitary	Globose to subglobse, thick-walled	4–9 × 6–10 µm
*Scedosporium aurantiacum*	Teleomorph unknown		Cylindrical or slightly flask-shaped	On branched coniodiophores or solitary	Obvoid, thick-walled	6–10 × 3–5 µm

## Data Availability

Not applicable. Invited review.
